# Structural Insights into the Globular Tails of the Human Type V Myosins Myo5a, Myo5b, and Myo5c

**DOI:** 10.1371/journal.pone.0082065

**Published:** 2013-12-10

**Authors:** Hana Velvarska, Dierk Niessing

**Affiliations:** 1 Institute of Structural Biology; Helmholtz Zentrum München – German Research Center for Environmental Health, Neuherberg, Germany; 2 Gene Center and Department of Biochemistry, Ludwig-Maximilians-University, München, Germany; National Research Council of Italy, Italy

## Abstract

Vertebrate type V myosins (MyoV) Myo5a, Myo5b, and Myo5c mediate transport of several different cargoes. All MyoV paralogs bind to cargo complexes mainly by their C-terminal globular domains. In absence of cargo, the globular domain of Myo5a inhibits its motor domain. Here, we report low-resolution SAXS models for the globular domains from human Myo5a, Myo5b, and Myo5c, which suggest very similar overall shapes of all three paralogs. We determined the crystal structures of globular domains from Myo5a and Myo5b, and provide a homology model for human Myo5c. When we docked the Myo5a crystal structure into a previously published electron microscopy density of the autoinhibited full-length Myo5a, only one domain orientation resulted in a good fit. This structural arrangement suggests the participation of additional region of the globular domain in autoinhibition. Quantification of the interaction of the Myo5a globular domain with its motor complex revealed a tight binding with dissociation half-life in the order of minutes, suggesting a rather slow transition between the active and inactive states.

## Introduction

Type V myosin (MyoV) motors move protein complexes, organelles, vesicles, and mRNAs along actin cables [1]. In contrast to protozoan and invertebrates, mammals have three MyoV paralogs, termed Myo5a, Myo5b, and Myo5c [2]. Although they share an almost identical domain organization, their expression patterns and cargo specificities show marked differences. Whereas Myo5a is most abundant in neuronal and skin cells [3], Myo5b is found in almost all cell types. Myo5c is mostly expressed in epithelial and glandular tissues [4] and its localization is clearly distinct from Myo5a or Myo5b [5]. A genetic disease called Griscelli syndrome (GS) type I has been linked to mutations in the *myo5a* gene [6]. Mutations in the *myo5b* gene of patients are associated with the microvillus inclusion disease [7], [8]. So far, no mutation in *myo5c* has been linked to a heritable syndrome.

The N-terminal, actin-binding head of MyoV consists of an ATP-dependent motor domain that transmits conformational changes from the active site of ATP hydrolysis to the calmodulin- or light chain-decorated lever arms [2]. This lever arm is followed by a coiled-coil rod, which mediates dimerization of the motor protein. The C-terminal globular domain (GD) defines this class of myosins and mediates the majority of cargo interactions [2].

High-resolution structures of GDs from type V myosins are available for Myo2p and Myo4p from budding yeast [9], [10]. Since pronounced differences exist in their overall shapes, it was suggested that this feature might directly contribute to their distinct cargo specificities. Given that also Myo5a, Myo5b, and Myo5c exhibit differential cargo preferences, such large differences in shapes might also exist in higher eukaryotes.

For cargo loading of MyoV motors, so called cargo adapters are needed. The perhaps largest known class of cargo adapters is Rab GTPases. They often interact directly with the MyoV GDs and thereby link the motor to membranes, vesicles and organelles. The so far best-studied transport event in higher eukaryotes is specific localization of melanosome organelles in melanocytes. Here, Myo5a binds to the adapter Melanophilin, which itself associates with the small Rab GTPase Rab27a [11] to move processively along actin filaments [12]. In absence of cargo binding and at low calcium concentrations, Myo5a no longer exists in an elongated, active state, but rather adopts a closed, autoinhibited conformation [13]–[15]. Autoinhibition of myosins and kinesins prevents futile hydrolysis of ATP in the cell. Melanophilin binding to the GD of Myo5a has been shown to reduce Myo5a autoinhibition *in vitro* [16]. To date, it is unclear whether Myo5b and Myo5c are also autoinhibited. For instance in yeast, the type V myosin Myo4p is monomeric in absence of cargo and is not autoinhibited by the canonical mechanism [17]–[19].

The approximate positioning of the GD into low-resolution electron microscopy reconstructions of murine Myo5a suggested that in this closed conformation the motor domain is placed in direct vicinity to the GD, forming an inactivated complex [15], [20]. Electron microscopic analysis of 2D crystals revealed a very similar arrangement [21]. However, due to the lack of a high-resolution structure of a globular domain from higher eukaryotes, its precise position with respect to the motor domain could not be determined and alternative structural models had been suggested [20]–[22]. Consequently, the deposited structural model of autoinhibited Myo5a contains all domains except for the globular domain (PDB-ID: 2DFS) and molecular details on the mechanism of autoinhibition remain speculative.

Biochemical studies with Myo5a confirmed that the globular domain indeed mediates autoinhibition [13]–[16], [23]. They also showed that residues K1706 and K1779 in the GD of mouse Myo5a and D136 in the motor domain are required for autoinhibition [23]. Because of the distance of D136 from the ATP-binding site, an allosteric inhibition had been proposed. Recent findings also suggest that calmodulin bound to the first IQ motif might also play a role in autoinhibition [24].

We show by SAXS measurements that the GDs of Myo5a, Myo5b, and Myo5c adopt very similar shapes in solution. We also solved the X-ray structures of GDs from human Myo5a and Myo5b and provide a homology model of the globular domain of Myo5c. Docking our Myo5a GD X-ray structure into low-resolution electron density of the Myo5a-autoinhibited state suggests binding of larger surface regions to the motor complex. Quantification of the interaction between motor and GD yielded nano-molar affinities and low dissociation rates, suggesting a rather slow transition between autoinhibited and active states of the motor protein.

## Results

### Globular domains of Myo5a, Myo5b, and Myo5C adopt similar shapes in solution

**Figure 1:**
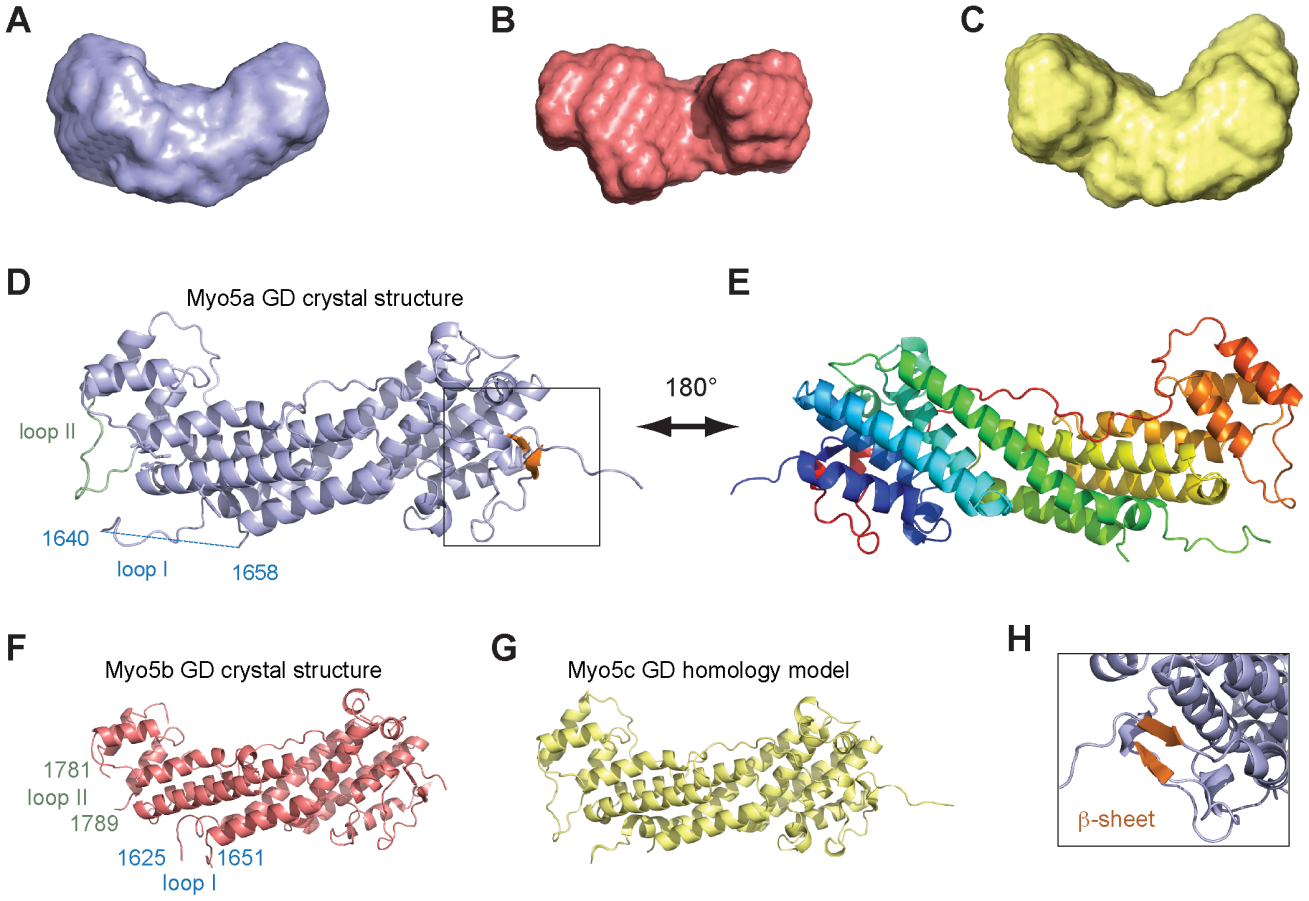
X-ray structures and Small-Angle X-ray Scattering (SAXS) models of globular domains from human type V myosins. (**A–C**) Surface envelopes calculated from SAXS data revealed that Myo5a GD (A), Myo5b GD (B), and Myo5c GD (C) have very similar shapes in solution. See Figure S1 in File S1 for details of SAXS measurements. (**D**) Ribbon representation of the Myo5a GD crystal structure at 2.2 Å, with the small C-terminal beta-sheet shown in orange. The missing loop I (1640 to 1658) is depicted as a blue dashed line, loop II is highlighted in green. (**E**) Same structure as in (D), but rotated 180° around the vertical axis. Rainbow color-coding follows the peptide chain from its N-terminus in blue to its C-terminus in red. (**F**) Ribbon representation of the Myo5b GD crystal structure at 3.1 Å resolution. First and last residue of the missing loops I and II are depicted in blue or green, respectively. (**G**) Ribbon representation of the modeled structure of the GD from Myo5c, calculated with the program Modeller and the structures of Myo5a (D–E) and Myo5b (F) as templates. For a Ramachandran plot of the computed model, see Figure S4 in File S1. (**H**) Close-up of (D), slightly rotated to better show the position of the small beta-sheet (orange) that connects the very N-terminus with the C-terminus. Figures were generated with the program Pymol.

Attempts to obtain protein crystals of fragments based on predicted domain boundaries [11] failed. Instead, limited proteolysis combined with mass-spectrometric analyses indicated that the GD is smaller and starts between amino acids 1446 and 1476. Indeed, a Myo5a fragment consisting of residues 1467–1855 (Myo5a (1467–1855)) was resistant to degradation and not prone to aggregation. Small Angle X-ray Scattering (SAXS) experiments with this and corresponding GD fragments of Myo5b and Myo5c showed that these fragments were monodisperse and clearly monomeric in solution (Figure S1 in File S1). The analysis also yielded calculated surface envelopes with similar overall shapes (Figures 1A-C and Figure S1 in File S1).

### The X-ray crystal structure of Myo5a globular domain

After several rounds of refinement, crystals were obtained with human Myo5a (1467–1855) that diffracted up to 2.5 Å. The structure was solved by multi-wavelength anomalous dispersion with selenomethionine-derivatized protein at 2.5 Å resolution (Table 1). Later, crystals were obtained with a different space group that diffracted up to 2.2 Å resolution. By performing molecular replacement with the derivatized structure as template an atomic model was built with this native dataset at a resolution of 2.2 Å (R_work_ = 0.22, R_free_ = 0.26; Table 1). The asymmetric unit contained two almost identical Myo5a molecules with a root mean square deviation for the c-α backbone atoms of 0.30 Å. Both molecules showed electron density for residues 1461 to 1855, with the exception of a predicted loop region ranging from residues 1640 to 1658 (loop I). The overall structural core of the Myo5a GD is hook-like shaped (Figures 1D,E) and its overall structural arrangement and connectivity is conserved in type V myosins (Figure S2 in File S1). It consists of 11 tightly packed α-helices followed by an extended region that clamps the entire structure like a bracelet. The peptide chain ends with a short helix, and a C-terminal two-stranded anti-parallel beta-sheet that folds against the very N-terminal residues (Figure 1H). Except for the beta-sheet, all of these elements are conserved in yeast homologs [9], [10].

**Table 1:** Data collection, phasing and refinement statistics for Myo5a globular domain (MAD).

	Native	Selenomethionine	
**Data collection**			
Space group	P2_1_2_1_2_1_	P3_2_21	
Cell dimensions			
* a*, *b*, *c* (Å)	74.06, 87.11, 130.94	146.92, 146.92, 200.00	
α, β, γ (°)	90, 90, 90	90, 90, 120	
		*Peak*	*Inflection*
Wavelength	0.9334	0.9785	0.9790
Resolution (Å)	43.5–2.2 (2.3–2.2)	49.3–2.5 (2.6–2.5)	49.3–2.5 (2.6–2.5)
*R*_sym_ or *R*_merge_	9.3 (48.3)	9.4 (25.6)	10.5 (31.3)
*I*/σ*I*	18.4 (5.0)	16.3 (7.5)	15.3 (6.4)
Completeness (%)	99.6 (99.5)	100 (99.3)	100 (94.9)
Redundancy	7.3 (7.5)	11.3 (11.5)	11.3 (11.5)
Phasing power		2.15 (for 48.0–2.8 Å)	
Figure of merit		0.48 (for 48.0–2.8 Å)	
**Refinement**			
Resolution (Å)	43.5–2.2	49.3–2.5	49.3–2.5
No. reflections	43,514		
*R*_work_/*R*_free_	21.5/25.6		
No. atoms			
Protein	6,493		
Water	427		
*B*-factors			
Protein	29.4		
Water	12.4		
R.m.s deviations			
Bond lengths (Å)	0.0136		
Bond angles (°)	1.04		

* Values in parentheses are for highest-resolution shell.

### The X-ray crystal structure of Myo5b globular domain

The structure of Myo5b GD was solved at 3.1 Å resolution by molecular replacement using Myo5a GD as template (Table 2). The asymmetric unit contains one Myo5b molecule (R_work_ = 0.25, R_free_ = 0.31), which shares with Myo5a the overall conservation of domain organization (Figure 1F). An overlay of both structures reveals a root mean square deviation (RMSD) of only 1.1 Å (Figure S2A in File S1). No electron density was visible for the N-terminal four amino acids, for loop I from amino acids 1625 to 1651 and for loop II (1781 to 1789) (Figure S3 in File S1).

**Table 2:** Data collection and refinement statistics for Myo5b globular domain (molecular replacement).

	Native
**Data collection**	
Space group	P2_1_2_1_2
Cell dimensions	
* a*, *b*, *c* (Å)	60.61, 78.33, 86.67
α, β, γ (°)	90, 90, 90
Resolution (Å)	49.7–3.1 (3.3–3.1)
*R*_sym_ or *R*_merge_	11.3 (68.3)
*I*/σ*I*	13.7 (2.4)
Completeness (%)	96.8 (93.6)
Redundancy	7.9 (8.0)
**Refinement**	
Resolution (Å)	49.7–3.1
No. reflections	7647
*R*_work_/*R*_free_	25.3/31.2
No. atoms	
Protein	2823
Water	0
*B*-factors	
Protein	58.0
Water	-
R.m.s. deviations	
Bond lengths (Å)	0.011
Bond angles (°)	1.428

* Values in parentheses are for highest-resolution shell.

### Structural similarities between MyoV homologs and homology model of Myo5c

A database search for homologous structures with the DALI server [25] confirmed the high structural similarities between the Myo5a (or Myo5b) GD and the yeast type V myosin homologs (Figures S2B,C in File S1). More limited structural homologies were observed to components of the membrane-tethering exocyst complex. Furthermore, the X-ray structures and SAXS surface envelopes presented here suggest that Myo5c GD might also adopt a similar fold. Myo5a and Myo5b GDs share 70.5% identical amino acids. Between Myo5a and Myo5c 62.7% of the residues are identical. Thus, the Myo5a and Myo5b GD structures serve as good templates for modeling an atomic structure of the Myo5c GD.

We performed sequence alignments between the three human type V myosins (Figure S3 in File S1) and calculated an atomic model of Myo5c GD (Figure 1G) using the program Modeller 9v7 [26]. The atomic model of Myo5c is compatible with the SAXS envelope (Figure 1C) and shows no obvious stereo-chemical abnormalities (Figure S4 in File S1). The model of the Myo5c GD has a very similar overall shape to Myo5a and Myo5b (Figures 1D–G) with RMSD values of only 1.1 Å between Myo5a and Myo5c (Figure S2D in File S1).

### Griscelli syndrome-causing mutations in the globular domain of Myo5a

Mutations in Myo5a that impair its function result in the type I Griscelli syndrome. Due to impaired Myo5a-dependent melanosome transport these patients exhibit partial albinism. One of the Griscelli mutations generates a short insertion at amino acid position 1545 with a premature stop codon in the globular tail [27]. Our structure of Myo5a shows that this truncation eliminates most of the GD (Figures S5A,B in File S1). We expressed this truncated fragment of Myo5a GD in fusion with a GST-tag and found it to be soluble. This finding suggests that this mutation does not cause protein degradation of Myo5a, but rather removes a functionally important region in the GD. However, it should be considered that this GD fragment might be less stable when N-terminally fused to Myo5a sequences instead of the GST-tag. Previously reported point mutations at residues I1512, M1515, and D1521 in the Myo5a GD [28], [29] are almost completely buried in the crystal structure (Figures S5C,D in File S1). Thus, these Griscelli mutations are likely to destabilize the GD.

### Comparison of surface features of the globular domains from Myo5a, Myo5b, and Myo5c

**Figure 2:**
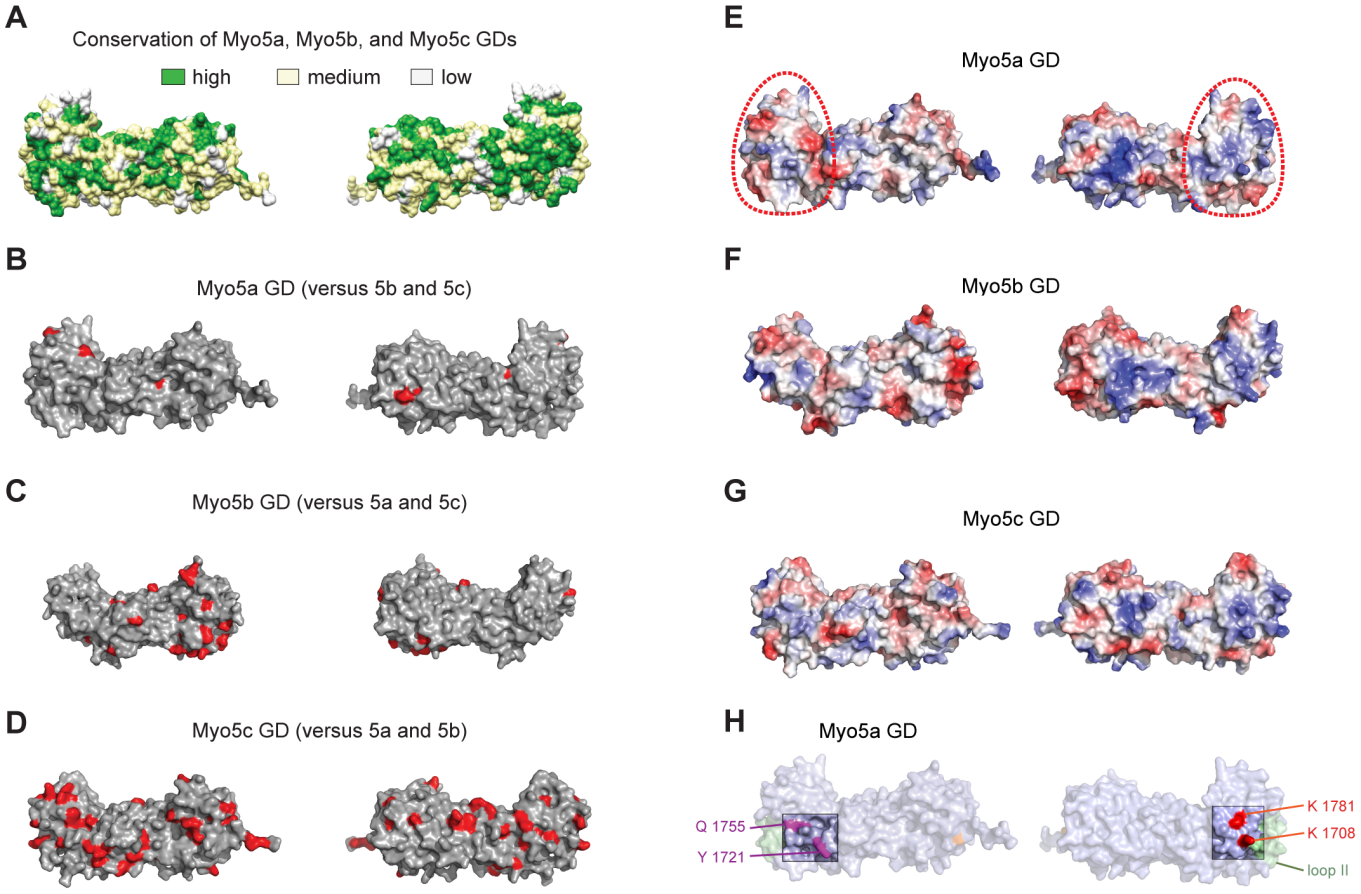
Analyses of surface properties of the globular domains from Myo5a, Myo5b, and Myo5c. Orientation is as shown in Figure 1D (left) and rotated by 180° around the vertical axis (right). (**A**) Amino acid conservation between human Myo5a, Myo5b, and Myo5c GDs based on alignment shown in Figure S3 in File S1 and plotted on the structure of Myo5a GD. Green indicates high sequence conservation, yellow partial conservation, and white a lack of conservation. (**B–D**) Unique surface residues in Myo5a (B), Myo5b (C), or Myo5c (D), when compared to their respective paralogs, are shown in red. (**E–G**) Representation of surface potentials of Myo5a (E), Myo5b (F), and Myo5c (G). Red and blue indicate surface areas with negative and positive surface charges, respectively. White regions indicate hydrophobic regions. The surface region encircled by a red dotted line is an area with high similarity of overall surface charges amongst the three type V myosins (see also Figure S6 in File S1). This similarity might hint at a common function in all three paralogs. (**H**) Amino acids required for Rab11a binding are highlighted in magenta, residues in red are essential for motor autoinhibition.

Since the overall shapes of Myo5a, Myo5b, and Myo5c are very similar, their reported differences in cargo specificity are likely due to distinct features on their surfaces. A surface plot of sequence alignments amongst the three MyoV GDs show a high degree of conservation (Figure 2A). Only few surface patches are unique in Myo5a (Figure 2B). A higher degree of deviation was observed for Myo5b (Figure 2C). The most distinct surface was observed for Myo5c (Figure 2D), suggesting that the latter has the most distinct interaction partners.

A surface plot of their electrostatic potentials revealed pronounced differences in certain regions, whereas other parts yielded similar charge properties (Figures 2E–G and Figure S6 in File S1). We found in all three paralogs a region covering almost one-third of the domain that shows very similar surface charge distributions (Figures 2E–G and Figure S6 in File S1, circled in red). This region could potentially mediate binding of an adaptor common to all three myosins or be required for a common function other than cargo binding. Indeed, we noticed that two amino acids important for autoinhibition in mouse Myo5a [23] fall within this highlighted region.

### Residues for autoinhibition are conserved in globular domains of all MyoV paralogs

**Figure 3:**
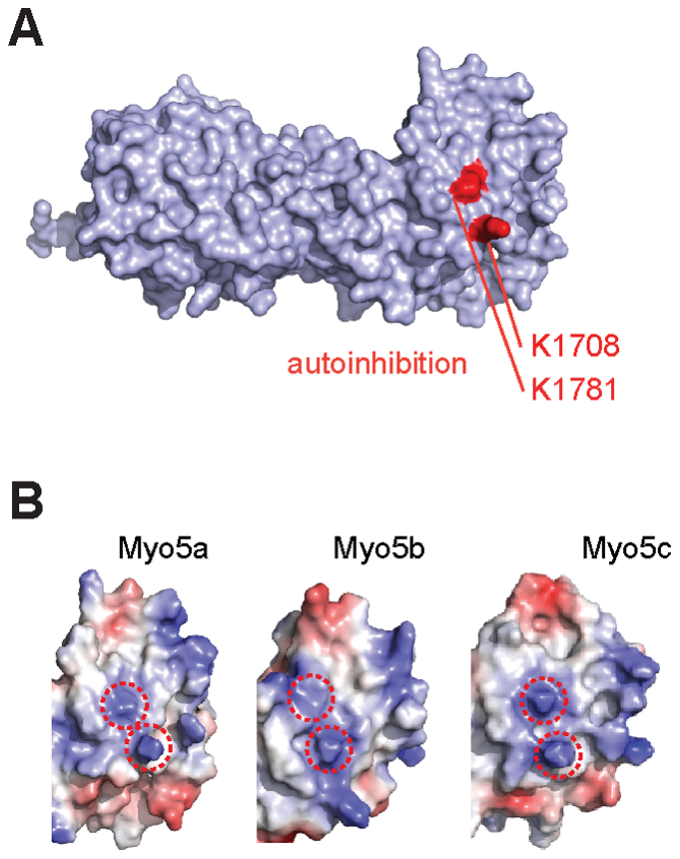
C-terminal globular domains of all three human type V myosins potentially mediate autoinhibition. (**A**) Surface representation of Myo5a GD with residues K1708 and K1781 highlighted in red. Their mutation resulted in a loss of autoinhibition [23]. (**B**) Comparison of electrostatic surface potentials of K1708 and K1781 (red circles) with identical positions in Myo5b and Myo5c and the conservation of these residues in all three paralogs (Figure S3 in File S1) suggests that autoinhibition might be a general feature of human type V myosins. Figures were generated with the program Pymol.

In human Myo5a, where autoinhibition has been observed, these residues correspond to K1708 and K1781. To date, no autoinhibition has been reported for Myo5b and Myo5c. However, these two residues are fully conserved in all three paralogs (Figure S3 in File S1; red-boxed amino acids) and are in the same position in Myo5a, Myo5b, and Myo5c (Figures 3A,B). This observation suggests that autoinhibition might not be limited to Myo5a.

### Docking of high-resolution Myo5a structure into EM density of autoinhibited Myo5a

**Figure 4:**
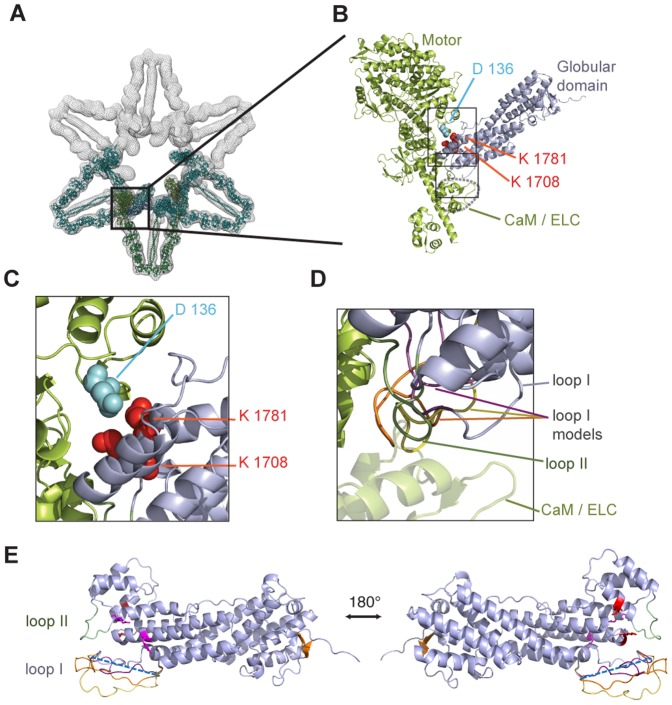
Autoinhibition of type V myosins by their globular domains involves multiple interactions sites. (**A**) Docking of our high resolution Myo5a GD structure into a previously published 24 Å electron density map of the inhibited Myo5a motor [21] (PDB-ID of published model lacking the globular domain: 2DFS). Shown are three modeled myosin dimers in the EM density (meshed surface rendering) that are arranged in a flower-like fashion. The color scheme is as follows: blue, Myo5a GD; green, motor domain, lever arm, light chains, coiled coil domain; turquoise, neighboring dimers. (**B**) Close-up of (A), depicting the Myo5a head complex (green) and the fitted GD (blue). Residues previously reported to be required for autoinhibition [23] are shown as colored spheres. (**C**) Close-up of the upper rectangle in (B). (**D**) Close-up of the lower rectangle in (B). Loop I is disordered in the structural data. Depicted are three computed models for the flexible loop I, highlighted in yellow, orange, and magenta. (**E**) Atomic model of Myo5a. Missing amino acids of the flexible loop I GD are depicted as a blue dashed line (disordered loop I: 1640–1658), loop II in green (1787–1797), and amino acids important for interaction with the motor domain are highlighted in red (K1708, K1781). Residues important for Rab11a binding (Y1721 and Q1755) are shown in magenta and the beta-sheet is depicted in orange. Computed models for the flexible loop I were depicted in yellow, orange, and magenta (see also D). Figures were generated with the program Pymol.

Because of the lack of a high-resolution structure of the human Myo5a GD, previous attempts to structurally understand the interaction between GD and motor domain of the autoinhibited full-length Myo5a was accompanied with uncertainty [20]–[22]. We therefore placed our high-resolution structure of Myo5a GD into the available electron density [21] by performing molecular dynamics flexible fitting (MDFF) [30]. In order to obtain an unbiased fit, the only constraint used in the fitting procedure was the superpositioning of our atomic model into the free electron density of the inhibited complex. In contrast to the previous models [20]–[22], only one orientation of the Myo5a GD could be fitted in a satisfactory manner (Figures 4A,B). In this orientation no changes in the structural conformation of the GD had to be modeled. Other orientations either resulted in serious clashes with the motor complex or gave large mismatches between the GD and free electron density. Similar to one of the previously suggested alternative orientations [20], [22], our fitting locates the residues K1708, and K1781 of the GD (Figure 3A) in close vicinity to D136 of the motor domain (Figures 4B,C). All three residues are required for autoinhibition [23].

Another potentially important feature in our fitted model is that loop I and II of the Myo5a GD (Figure 4E and Figure S3 in File S1) are positioned close to the motor complex (Figures 4B,D). Because no electron density was observed for loop I in our crystal structure, we modeled possible conformations of the loop (Figure 4D, E). Several of these spatial conformations were in close proximity to the lever arm and calmodulin (Figure 4 B, D). Also the position of loop II was close enough for potential contacts with the head complex (Figure 4D). It is therefore possible that additional surface regions of the GD participate in the autoinhibited complex. However, to understand these features, an experimental high-resolution co-structure would be required.

### Quantification of the interaction between Myo5a motor complex and its globular domain

**Figure 5:**
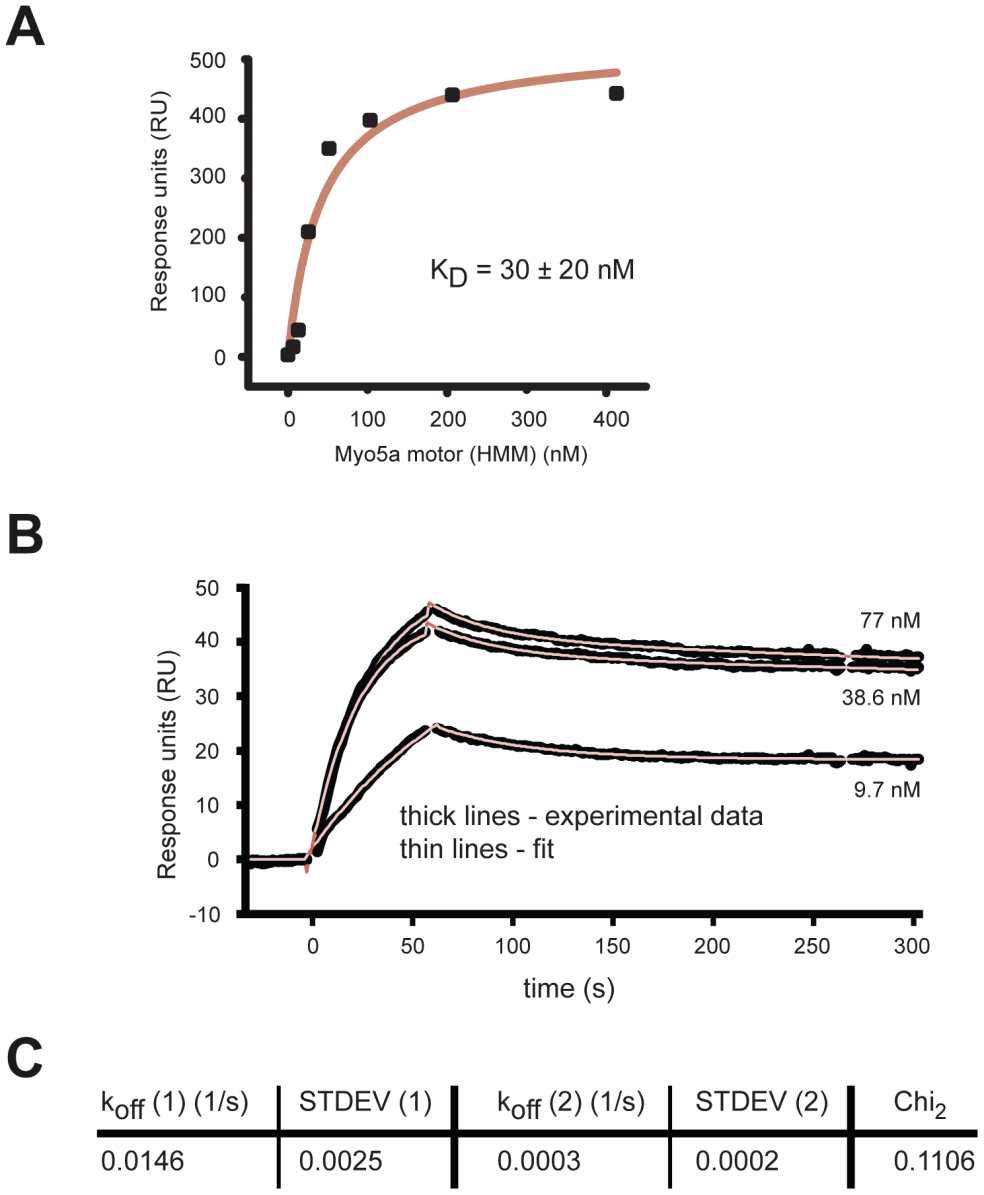
Surface plasmon resonance (SPR) measurements with surface-coupled Myo5a globular domain (GD) and the motor complex. (**A**) Diagram shows a representative steady-state binding experiment with surface-coupled Myo5a GD and its motor complex (Myo5a HMM) using a multi-injection protocol. The K_d_ = 30±20 nM was derived from two independent measurements, as recommended by the manufacturer. (**B, C**) Kinetic binding experiments of Myo5a GD interaction with its motor complex (Myo5a HMM). (B) shows sensograms with representative kinetic measurements (thick lines) at 77 nM, 38.6 nM, and 9.7 nM Myo5a (HMM) and the corresponding curve fittings (thin lines). Off-rates were determined from this concentration range using bivalent curve fitting. Curve fittings with chi_2_<0.2 (n = 5) were used to determine average K_off_ values (C).

Although the interaction between the motor domain of Myo5a and its C-terminal tail has been studied in detail, no direct quantification is available for this binding event. To obtain equilibrium dissociation constants (k_d_), we used surface-plasmon resonance with surface-coupled Myo5a GD. We first performed binding experiments under steady-state conditions. The murine Myo5a HMM complex bound to the GD with a k_d_ = 30±20 nM (Figure 5A). In order to assess the stability of this interaction, we subsequently performed kinetic binding experiments and determined off-rates (Figure 5B). The deduced k_off_ values show that the dissociation of this interaction is a slow event (Figure 5C), suggesting that the switch between inactive, autoinhibited to active state takes place in the order of minutes.

## Discussion

The GD of MyoV motors plays a central role in cargo binding for their transport. In vertebrate Myo5a, the GD also mediates autoinhibition of ATPase activity in its cargo-free state. Whether Myo5b and Myo5c undergo autoinhibition is unknown to date. Furthermore, no high-resolution structure existed for GDs of higher eukaryotes. The GDs of yeast Myo2p and Myo4p have similar domain architectures, but different overall shapes [9], [10]. Hence, it had been speculated that cargo specificity might be linked to such shape differences. Similar differences in shape could potentially also be responsible for diverging cargo specificities of human MyoV motors.

In this study, we describe the high-resolution X-ray structures of the GDs from human Myo5a and Myo5b as well as low-resolution SAXS-surface envelopes for all three human MyoV paralogs. Surprisingly, the shapes between GDs from human Myo5a, Myo5b, and Myo5c are almost identical. Their ability to bind different cargoes must therefore mainly rely on their surface properties.

We further found that two residues known from yeast MyoV to be important for auto-inhibition are conserved at specific surface sites in the GDs of all three human paralogs. A second feature required for autoinhibition in Myo5a is the existence of a flexible hinge region (amino acids 1238 to 1337) splitting the coiled-coil. This hinge is essential for adopting the autoinhibited state of the motor (Figure 4A). It is therefore interesting that also in Myo5b and Myo5c the coiled-coil region is separated by sequence stretches of 74 and 40 residues, respectively, with low coiled-coil probability. These hinge regions contain sequence stretches with amino acid identities to the Myo5a hinge in the range of 30% to 58%. In summary these findings suggest that all three type V myosin paralogs may be able to undergo autoinhibition.

During submission of this manuscript the crystal structure of the Myo5a GD from mouse was published [31]. While there are differences in resolution between the mouse structure (2.5 Å) and our human structure (2.2 Å), the overall structural features are very similar. Like in our structure, the very N- and C-termini of the GD form a joint beta-sheet and electron density for the same loop region is missing in both structures. Wei and colleagues also solved the structure of a ternary complex consisting of Myo5a GD, the RH1 domain of the Rab interacting lysosomal protein-like 2, and a short fragment of melanophilin [31]. All interactions between these molecules occur in the beta-sheet containing half of the GD (Figure 1D). Because the residues required for auto-inhibition are located on the other side of the GD (Figure 3A,B), it seems unlikely that these interactions directly interfere with the autoinhibition of Myo5a. We also noticed that residues for Rab11a binding are located on a surface region far away from residues important for autoinhibition (Figure 2H, 3A).

Fitting our high-resolution X-ray structure of Myo5a GD into the low-resolution electron density of autoinhibited Myo5a [21] allowed for only one possible orientation of the GD. In contrast, previously reported low-resolution orientation of the GD into density of the inhibited motor had suggested more that one conformation [20]–[22]. Our docking of the Myo5a GD in the EM density positioned its previously reported interacting residues and two loop regions in a triangular arrangement, consisting of the motor domain, the lever arm and calmodulin. Thus, our structural docking suggests that in addition to the previously described three conserved amino acids [23] other surface regions of the GD participate in autoinhibition of the motor complex.

Available EM and sedimentation centrifugation studies consistently showed that the closed, autoinhibited state does not require actin binding [13], [14], [20], [21]. When unbound from actin, the motor domain freely rotates about the hinge between motor and lever arm [32], [33]. Interestingly, all autoinhibited motor domains have been found in a defined orientation that is most compatible with the post-power stroke state. This fixed orientation is in stark contrast to the reported flexibility of the active motor. Also crystallographic and EM studies showed a variety of different orientations of the free motor [34]. It can be assumed that GD binding to the motor is responsible for the reduced flexibility of autoinhibited Myo5a. Our quantification of the motor-GD interaction yielded a k_d_ in the nanomolar range and slow dissociation rates. These binding properties are likely to contribute to this fixed, inactive state of MyoV. Whereas one of these k_off_ values suggests disassembly of the autoinhibited state in the range of minutes, the second k_off_ values indicates complex half-lifes in the range of hours. Because such very stable inactive complexes are difficult to imagine in a cellular context, motor activation must either be a process involving an activation step or the measured values have to be attributed to interactions of the myosin motor with the chip surface. In contrast, the off-rate in the range of minutes seems to fit well with its biological function. Thus the low dissociation rates further suggest that the transition from the inactive to the active state is not a fast oscillating event.

## Materials and Methods

### Protein purification

Globular domain (GD) fragments of human Myo5a (1467–1855), Myo5b (1462–1849), and Myo5c (1355–1742) were cloned into pGEX-6P-1 vectors (GE Healthcare), Expression and purification was performed according to standard protocols [35]. The GST tag was cleaved and removed. For final structural analyses, the fragments Myo5a (1467–1855), Myo5b (1462–1849), and Myo5c (1355–1742) were used. Selenomethionine-substituted Myo5a GD was expressed in DH10 cells grown in selenomethionine containing medium and purified as described in presence of 20 mM DTT. Myo5a (HMM) is a fragment of murine Myo5a (1–1105) C-terminally fused to GCN4-Flag. HMM was coexpressed with essential light chains and calmodulins in *Spodoptera frugiperda* Hi5 insect cells using the MultiBac multigene vector system [36] and purified according to [14]. Murine calmoduline was cloned into pET28a with NcoI and NheI for expression without a tag. After expression in *E. coli*, the protein was purified with HiTrap Phenyl sepharose (GE Healthcare) and a size exclusion chromatography column.

### Crystallization and structure determination

Crystals of Myo5a GD were grown at 21°C by hanging-drop vapor-diffusion using 1∶1 mixture of protein (1 mg/ml) and crystallization solution containing 0.112–0.125 M succinic acid pH 7.0, 16–18% PEG 3350 for native crystals and 100 mM MES pH 5.5, 6% dioxane, 1.45 M (NH_4_)_2_SO_4_, 10 mM DTT for selenomethionine (SeMet) substituted crystals. After several rounds of optimization and refinement, crystals appeared within 2–3 days. Native crystals were cryoprotected in 10% ethylene glycol, SeMet crystals in 20% glycerol. Crystals of Myo5b were grown at 18°C by hanging-drop vapor-diffusion using 1∶1 mixture of protein (0.8 mg/ml) and crystallization solution containing 50 mM Tris pH 7.0, 200 mM succidic acid and cryoprotected with 25% glycerol. Each dataset was recorded from a single crystal at the following beamlines of the European Synchrotron Radiation Facility: Myo5a Native: ID14-1; Myo5a SeMet: ID14-4; Myo5b Native: ID23-1. Datasets were integrated and scaled using XDS [37] and Scala [38]. Since attempts to solve the crystal structure of Myo5a GD by molecular replacement with yeast homologs (Myo2p, PDB-ID: 2F6H or Myo4p PDB-ID: 3MMI) as templates failed, the structure was solved by multiwavelength anomalous dispersion (MAD) with SeMet substituted crystals at 2.5 Å resolution (Table 1). 84 selenium atoms were located using HKL2MAP/SHELXD program [39] and phases obtained with SHARP [40]. A native dataset of Myo5a crystals was used to perform Molecular Replacement with the program PHASER [41] and the SeMet-derived model as template to improve the resolution to 2.2 Å (Table 1). After partial automatic model building with Arp/wARP [42], further manual model building was carried out with COOT [43]. Refinement was performed using Phenix [44]. The final model was analyzed using SFCHECK [38]. The structure of Myo5b GD was solved by Molecular Replacement with the Myo5a GD structure as template (Table 2) using the program PHASER. Protein Databank ID for Myo5a GD and for Myo5b GD are 4LLI and 4LNZ, respectively.

### Small-angle X-ray scattering (SAXS)

SAXS measurements were carried out at beamline ID 14-3 (European Synchrotron Radiation Facility, France), at a wavelength of 0.931 Å. Various concentrations of protein samples (from 1.1 to 14 mg/ml) in 50 mM Tris pH 7.0 (or pH 7.5 for Myo5b and Myo5c GD), 140 mM NaCl and 2 mM DTT were exposed 10 times for 30 s at 293 K. Each data trace was subjected to a linear fit in the Guinier region (*Q* ^2^<0.013 Å^−2^) to determine the values of the slope (−*R*_g_^2/3^) and the *y* intersection {ln[*I*(O)]}. Intrinsic values of I(O)/concentration and *R*_g_ were estimated by linear extrapolation to zero concentration. Using a reference of BSA [45], the scattering intensities were extrapolated to zero angle I(0). The pair correlation function *P*(r) was calculated using the GNOM program package. Bead models were calculated and averaged with GASPORp. The *P*(r) functions of the model were obtained from the crystal structures were calculated using CRYSOL and GNOM [46].

### Surface plasmon resonance experiments

Surface plasmon resonance analysis was performed at 25°C on a Biacore 3000 system in running buffer (20 mM MOPS-KOH pH 7.0, 0.1 M NaCl, 2 mM MgCl_2_, 0.5 mM EGTA, 10 µM Calmodulin, 1 mM TCEP). Immobilization of Myo5a GD (1467–1855) was performed according to manufacturer's instruction. Briefly, the dextran surface of the CM5 chip was activated by a 1∶1 mixture of 50 mM N-hydroxysuccinimide and 0.2 M 1-ethyl-3-(3-dimethyl-aminopropyl)-carbodiimide hydrochloride. Myo5a GD in 10 mM sodium acetate pH 5.5 was injected to the activated surface. Excess of reactive groups was deactivated by 1 M ethanolamine hydrochloride-NaOH (pH 8.5). A density of about 50–100 resonance units (RU≈pg/mm^2^) of covalently immobilized Myo5a GD was obtained (n = 2). The same procedure was used for a blank surface, except for the injection of Myo5a GD protein. For single cycle kinetics measurements, HMM was injected at a flow rate of 30 µl/min for 170 s (0 – 413 nM). For dissociation constant measurement, HMM was injected at a flow rate of 30 µl/min for 60 s at three different Myo5a HMM concentrations at 9.7 nM, 38.6 nM and 77 nM as randomized duplicates (n = 5). Values of *K*_D_ and k_off_ are reported as the means of independent experiments with corresponding standard deviations. k_on_ values followed higher order kinetics, for which fits could be obtained. However, we were not confident that these fits are meaningful. Hence, no k_on_ values are reported.

### Bioinformatics

Sequence alignment was performed with ClustalW [47], coiled-coil prediction with COILS [48], structural superpositioning with SSM [49] using COOT [43], calculation and representation of the electrostatic surfaces and images of the crystal structures with Chimera [50], Swiss PDB Viewer [51] and Pymol [52].

## Supporting Information

File S1 **Contains the following figures: Figure S1** Small Angle X-ray Scattering (SAXS) experiments with the C-terminal globular domains of human Myo5a, Myo5b, and Myo5c. **Figure S2** Superpositioning of globular domains of type V myosins from yeast and humans. **Figure S3** Alignment of protein sequences from the globular domains of human Myo5a, Myo5b, and Myo5c. **Figure S4** Ramachandran plot of the homology model for the Myo5c globular domain. **Figure S5** Mutations found in patients with Griscelli syndrome that map to the globular domain of Myo5a. **Figure S6** Electrostatic potentials of Myo5a, Myo5b, and Myo5c calculated with the program Swiss PDB viewer.
(PDF)
