# Supplementary material for: Structural Insights into the Globular Tails of the Human Type V Myosins Myo5a, Myo5b, and Myo5c
Source: PLoS One. 2013 Dec 10;8(12):e82065. doi: 10.1371/journal.pone.0082065 (PMC3858360; doi:10.1371/journal.pone.0082065)
Supplement: File S1 — Contains the following figures: Figure S1 Small Angle X-ray Scattering (SAXS) experiments with the C-terminal globular domains of human Myo5a, Myo5b, and Myo5c. Figure S2 Superpositioning of globular domains of type V myosins from yeast and humans. Figure S3 Alignment of protein sequences from the globular domains of human Myo5a, Myo5b, and Myo5c. Figure S4 Ramachandran plot of the homology model for the Myo5c globular domain. Figure S5 Mutations found in patients with Griscelli syndrome that map to the globular domain of Myo5a. Figure S6 Electrostatic potentials of Myo5a, Myo5b, and Myo5c calculated with the program Swiss PDB viewer. (PDF) [file pone.0082065.s001.pdf]

Supporting Figures

A

	Conc. (mg/ml)	Conc. (µM)	Rg (nm)	Dmax (nm)	MW by SAXS (kDa)	Theor. MW (kDa)	Square root of chi-square
Myo5a GD	1.1<br>1.3<br>3.0	24.4<br>28.9<br>66.7	3.24<br>3.08<br>3.12	11.1<br>10.8<br>10.9	51<br>59<br>45	45.0	3.74
Myo5b GD	1.6<br>2.2<br>2.5	35.6<br>48.9<br>55.6	3.20<br>3.17<br>3.12	10.2<br>11.1<br>10.5	52<br>44<br>39	44.9	1.86
Myo5c GD	6.1<br>10.0<br>14.1	135.6<br>222.2<br>313.3	3.23<br>3.29<br>3.17	11.9<br>11.6<br>11.0	37<br>39<br>49	45.1	----

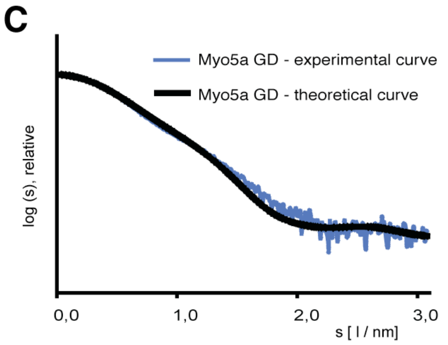

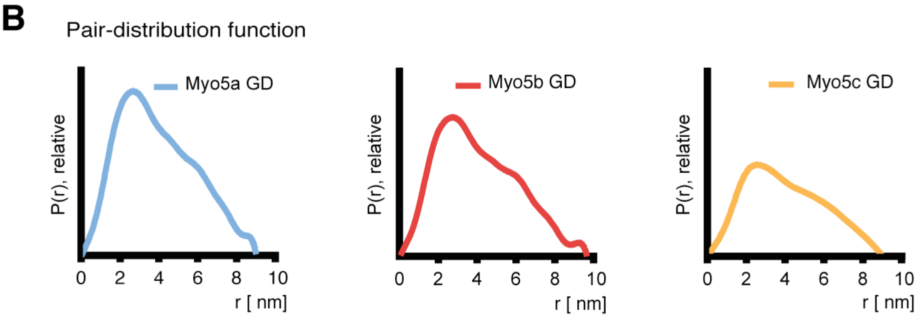

Figure S1 Small Angle X-ray Scattering (SAXS) experiments with the C-terminal globular domains of human Myo5a, Myo5b, and Myo5c. **(A)** Table summarizing SAXS experiments. The data show that the overall dimensions of all three paralogs are very similar and that they all exist as monomers in solution. Chi-square values indicate how good the fits of the atomic structures are for the SAXS envelopes. Because for Myo5c no experimental structural model exists, also no chi-square value was calculated. **(B)** Pair-distribution function for each globular domain, as used for the calculation of surface envelopes (Figures 1A-C). **(C)** Overlay of the experimental scattering curve of Myo5a GD (blue) and the theoretical scattering curve (black) calculated from the Myo5a GD crystal structure presented in this study. The very good overlay of both curves confirms the accuracy of the surface envelope of Myo5a (Figure 1). The calculation of the theoretical scattering curve was done with the program CRYSOL.

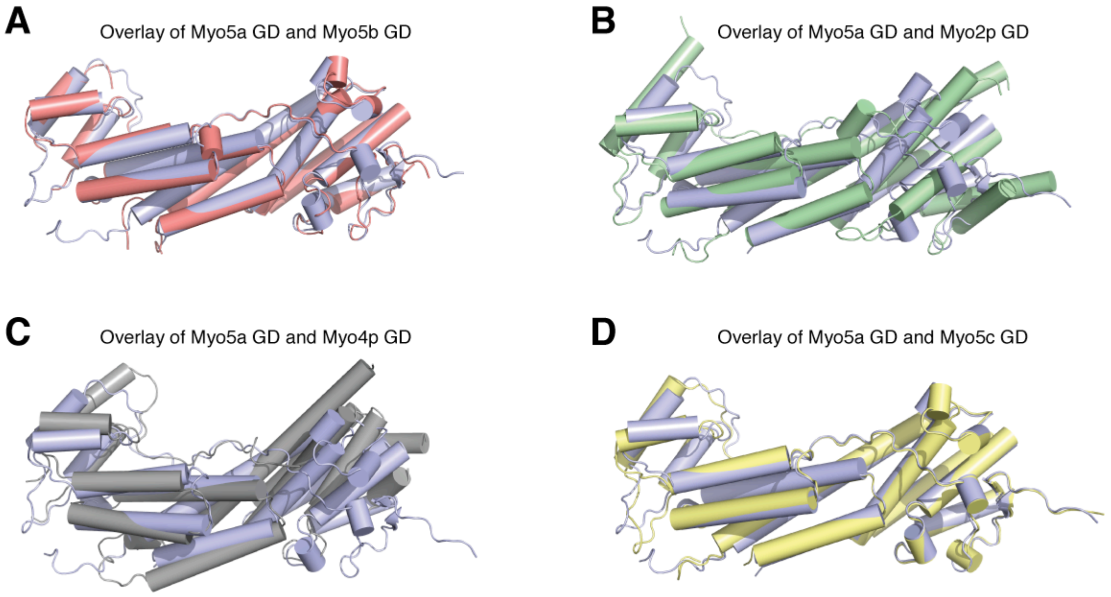

Figure S2 Superpositioning of globular domains of type V myosins from yeast and humans. **(A)** Overlay of the experimental structures of human Myo5a GD (blue) and Myo5b GD (red) (RMSD = 1.1 Å). **(B)** Overlay of human Myo5a GD (blue) and yeast Myo2p GD (green) (PDB-ID: 2F6H; RMSD = 2.4 Å). **(C)** Overlay of human Myo5a GD (blue) and yeast Myo4p GD (grey) (PDB-ID: 3MMI; RMSD = 3.2 Å). **(D)** Overlay of human Myo5a GD (blue) with the homology model of human Myo5c GD (yellow) (RMSD = 1.1Å). The model of Myo5c GD was generated with the structures of Myo5a GD and Myo5b GD as templates. Superpositioning was performed using COOT and ss-superimpose.

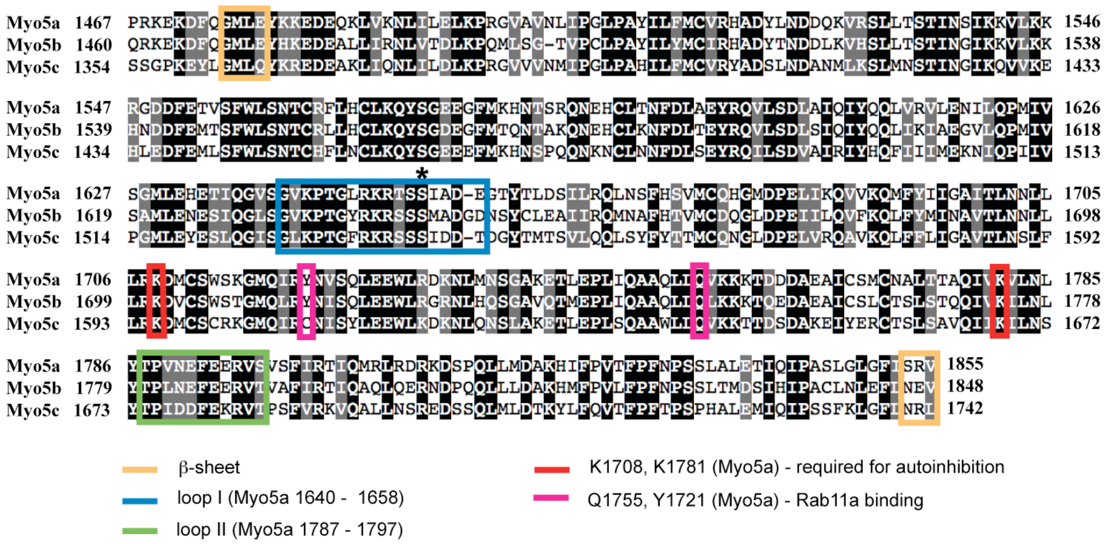

Figure S3 Alignment of protein sequences from the globular domains of human Myo5a, Myo5b, and Myo5c. Identical amino acids are highlighted by black background, grey background indicates moderate conservation and white means low conservation. Colored boxes show functional or structural regions (see legend) that are discussed in the main text. Asterisk indicates the previously reported phosphorylation site serine 1652 [1]. Color-coding of boxed amino acids is identical with Figs. 3A,C, and 4E. Alignment was generated with the program Clustal.

1. Karcher RL, Roland JT, Zappacosta F, Huddleston MJ, Annan RS, et al. (2001) Cell cycle regulation of myosin-V by calcium/calmodulin-dependent protein kinase II. Science 293: 1317-1320.

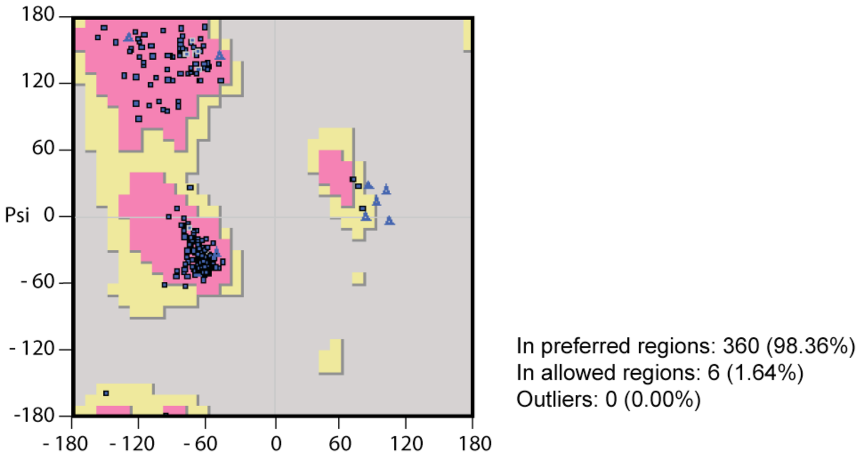

Figure S4 Ramachandran plot of the homology model for the Myo5c globular domain.

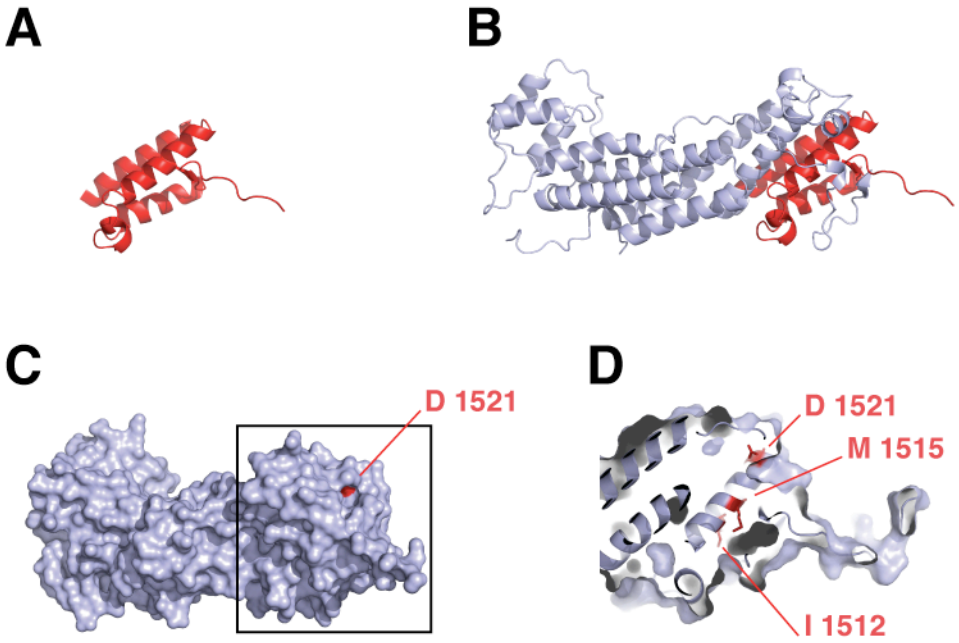

Figure S5 Mutations found in patients with Griscelli syndrome that map to the globular domain of Myo5a. **(A)** Fragment of the Myo5a globular domain that is expressed in a Griscelli patient (amino acids 1467-1545). **(B)** The same fragment (red) in context of the full Myo5a GD (blue). **(C)** Mutation of three individual amino acids (I1512, M1515, D1521) were found in mice to abolish binding to the cargo adapter melanophilin and thus may also cause Griscelli syndrome in humans. A surface representation of Myo5a GD shows that only D1521 is exposed with a very small patch to the solvent (shown in red). Thus, this and the other mutations are likely to induce the reported defects by impairing the overall domain fold of the GD. **(D)** Close-up of boxed region in (C) as a combined surface and ribbon representation. I1512, M1515 and D1521 are highlighted in red.

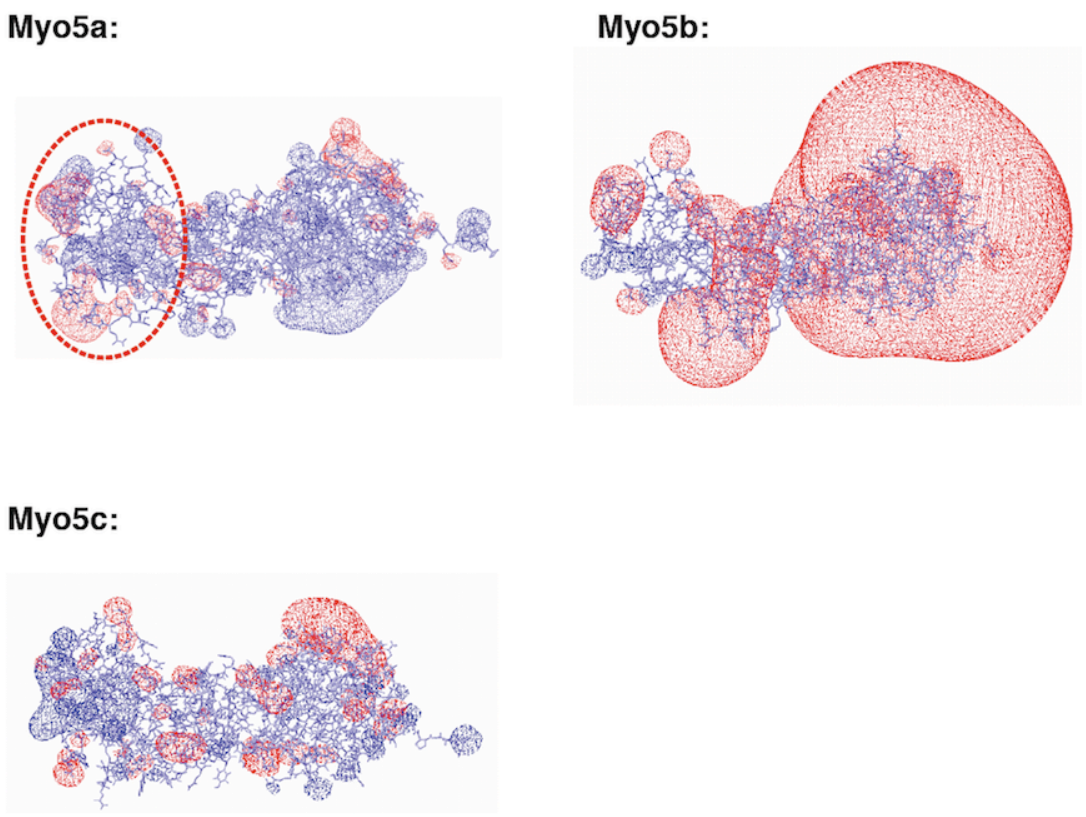

Figure S6 Electrostatic potentials of Myo5a, Myo5b, and Myo5c calculated with the program Swiss PDB viewer. The larger the cloud appears around a certain region, the greater its joint electrostatic potential is. Positive charges are depicted in blue, negative charges in red. Please note that the region of high similarity highlighted in Figure 2E also shows high similarity in this plot (region of Myo5a encircled with dotted line). For each image identical parameters were used.
